# Effect of implementation of a clinical checklist on the evaluation of constitutional mismatch repair deficiency in a neurofibromatosis clinic

**DOI:** 10.1007/s12687-026-00967-8

**Published:** 2026-10-30

**Authors:** Bridget McDonald, Jennifer Ivanovich, Leah Wetherill, Laurence Walsh, Jayne Vonbergen, Lisa Langsford, Steven D. Rhodes, Leigh Anne Stout

**Affiliations:** 1 Department of Medical and Molecular Genetics, Indiana University School of Medicine, Indianapolis, IN USA; 2 Indiana University Health Physicians, Indianapolis, IN USA; 3 Division of Pediatric Neurology, Indiana University School of Medicine, Indianapolis, IN USA; 4 Division of Pediatric Hematology/Oncology/Stem Cell Transplant, Indiana University School of Medicine, Indianapolis, IN USA; 5 Indiana University Simon Comprehensive Cancer Center, Indiana University School of Medicine, Indianapolis, IN USA; 6 Department of Pediatrics, Herman B Wells Center for Pediatric Research, Indiana University School of Medicine, Indianapolis, IN USA; 7 IU Health Simon Cancer Center, 1030 W. Michigan St, Ste 3307, Indianapolis, IN 46202 USA

**Keywords:** Genetic, CMMRD, Neurofibromatosis, Checklist, Genetic testing

## Abstract

**Background:**

Children with constitutional mismatch repair deficiency (CMMRD) require early and intensive cancer surveillance. Due to phenotypic overlap between CMMRD and neurofibromatosis type 1 (NF1), some children with CMMRD receive an incorrect diagnosis of NF1 and are inappropriately managed for their cancer risk. This study evaluated the effectiveness of a clinical checklist in identifying individuals for whom CMMRD evaluation is recommended.

**Procedure:**

Providers in a neurofibromatosis clinic implemented published CMMRD evaluation checklists into their clinical notes. Phase 1 of the study consisted of retrospective chart review prior to incorporation of the checklist. Phase 2 consisted of prospective chart review after checklist implementation.

**Results:**

Phase 1 included 81 patients; Phase 2 included 60 patients. In Phase 2, ambiguous (“Unknown”) checklist responses were reduced, and patients were more likely to meet criteria for CMMRD evaluation. Individuals in Phase 2 were more likely to be referred for genetic evaluation compared to Phase 1. There was no correlation between patients meeting criteria for CMMRD evaluation and being referred to genetics in either cohort.

**Conclusions:**

These results suggest a clinical CMMRD checklist is a useful tool for identifying individuals for whom CMMRD evaluation is recommended. Patients with a nondiagnostic NF1 workup who meet CMMRD evaluation criteria require further follow-up to aid in appropriate diagnosis and management.

**Supplementary Information:**

The online version contains supplementary material available at https://doi.org/10.1007/s12687-026-00967-8.

## Introduction

Constitutional mismatch repair deficiency (CMMRD) is a rare, autosomal recessive cancer predisposition syndrome caused by biallelic pathogenic variants (PVs) in one of the four mismatch repair (MMR) genes *– PMS2*,* MSH6*,* MLH1*, or *MSH2* (Suerink 2019). These genes correct DNA replication errors, and biallelic inactivation eliminates this repair capacity, predisposing affected individuals to multiple malignancies beginning in childhood. Individuals who carry a single PV in a DNA MMR gene instead have Lynch syndrome, a hereditary cancer syndrome that predisposes to colon, uterine, gastric, and other cancers. Many Lynch syndrome-associated cancers are associated with earlier age of onset and cancer risks vary depending on the causative gene. Therefore, both parents of a child with CMMRD, each with a PV in one of the MMR genes, have Lynch syndrome (Urganci 2015), highlighting the need to perform a detailed pedigree assessment.

The most common CMMRD-associated cancers are central nervous system (CNS) tumors, gastrointestinal cancers, and hematological malignancies (Ercan 2024). Rhabdomyosarcoma, Wilms tumor, and female reproductive tract cancers have also been reported (Bush 2019, Wimmer 2008). Nearly all affected individuals develop cancer in childhood or adolescence (Aronson 2022), with one study reporting malignancies in 96% (22 of 23) individuals aged 3 to 23 (Bakry 2014). Survivors of first malignancies remain at high risk for developing subsequent primary malignancies (Wimmer 2014). Intensive surveillance is recommended to begin at the time of diagnosis (Colas 2024, Das 2024). CMMRD also has a non-tumor phenotype, including multiple café-au-lait macules (CALMs) and intertriginous freckling. These cutaneous manifestations are commonly seen in neurofibromatosis type 1 (NF1), often making a diagnosis of CMMRD difficult. NF1 is an autosomal dominant disorder affecting approximately 1 in 3,000 people (Lee 2023). It arises from heterozygous loss-of-function variants in the *NF1* gene and increases the risk of CNS tumors, malignant peripheral nerve sheath tumors (MPNST), and female breast cancer (Uusitalo 2016). The risk of these cancers is significantly lower than in individuals with CMMRD. Clinical manifestations of NF1 include multiple café-au-lait macules (CALMs), axillary and/or inguinal freckling, cutaneous and plexiform neurofibromas, gliomas, and Lisch nodules (Legius 2021). In addition to the cutaneous findings common to both CMMRD and NF1, CMMRD is rarely associated with some of these features, including neurofibromas, gliomas, and Lisch nodules, further complicating the distinction between these disorders (Bakry 2014, Wimmer 2017). It is estimated that 1 in 258 children suspected of having NF1, who test negative for pathogenic *NF1* variants, instead have CMMRD (Suerink 2019). Medical management and surveillance guidelines vary significantly between these two syndromes; thus, establishing an accurate diagnosis is crucial. Additionally, clarifying the correct diagnosis has important implications for genetic counseling as the sibling recurrence risk for CMMRD is 25%, compared to less than 1% for a de novo NF1 PV (Wimmer 2014, Radtke 2007).

Wimmer et al. (2014) and Suerink et al. (2019) published criteria for CMMRD evaluation and testing for children with and without malignancy at the time of initial presentation, respectively. Hicks et al. (2021) performed a retrospective chart review of individuals seen in neurofibromatosis specialty clinics and found that 55% (133 of 240) individuals with features of NF1 had not undergone genetic testing. Among those tested, 23% (25 of 107) had nondiagnostic results, including 22 individuals who tested negative for both *NF1* and *SPRED1* variants and 3 individuals with an *NF1* variant of uncertain significance (VUS). In a cohort of 735 patients, Perez-Valencia et al. (2020) identified three cases (0.41%) of CMMRD in individuals with NF1-like features and negative *NF1/SPRED1* genetic testing. Collectively, these findings support the consideration of additional genetic evaluation for CMMRD for individuals with clinical features of NF1 who do not have a molecular diagnosis. The high rate of early onset malignancy associated with CMMRD makes timely detection critical.

This study explored whether embedding published CMMRD evaluation criteria in the clinical notes of neurofibromatosis providers, including a pediatric oncologist, a pediatric neurologist/geneticist, and a nurse practitioner, prompted more systematic evaluation for CMMRD.

## Methods

### Research design

We performed a retrospective chart review to determine whether adding a standardized CMMRD evaluation checklist to clinicians’ notes in the Neurofibromatosis (NF) Program at Riley Hospital for Children at Indiana University Health improved documentation and referral practices. Medical records were reviewed for patients seen prior to checklist implementation (Phase 1) and after the checklist was introduced (Phase 2). The study was deemed exempt research under Category 4(iii) by the Indiana University Institutional Review Board (Protocol number: 25199) and the Indiana University Melvin and Bren Simon Cancer Center Scientific Review Committee (Protocol number: IUSCCC-25199).

### Participants

Participants were identified from the NF Program’s internal roster of individuals evaluated for suspected or confirmed NF1. The first author (BM) systematically reviewed each patient’s medical record using a standardized protocol, and two genetic counselors (coauthors JI and LAS) conducted an initial concordance review of 10 records to ensure consistency and accuracy of data extraction. A brief chart review was performed to confirm genetic test results for individuals with a known pathogenic or likely pathogenic (P/LP) *NF1 or SPRED1* variant. Patients diagnosed with or suspected of having *NF2*-related schwannomatosis were excluded.

### Study phases

To evaluate the impact of the checklist, we defined two cohorts. The pre-implementation cohort (Phase 1), included all patients evaluated by one of three participating providers in the NF Program between October 1, 2023, and October 31, 2024, prior to checklist implementation. The post-implementation cohort (Phase 2), comprised patients evaluated by one of the same three participating providers who completed the checklist in their clinical note between November 1, 2024, and August 12, 2025. If a patient was seen more than once within the same phase, the most recent clinic note within that period was reviewed. The checklist was integrated into the electronic medical record (EMR) that providers completed during the encounter.

### Instrumentation

To standardize data collection, we embedded published CMMRD evaluation criteria into two structured electronic checklists (See Supplemental Materials). Patients with a prior history of malignancy were assessed using the 16-item “Malignancy checklist” derived from the criteria of Wimmer et al. (2014). Although the Wimmer et al. (2014) checklist for patients with cancer was revised by Colas et al. (2024)after the start of our study, this checklist was only used for 3 patients and statistical analyses were not performed on this group given the sample size. Colas et al. (2024) made no revisions to the Suerink et al. (2019) guidelines for patients without cancer. Patients without prior history of malignancy were evaluated using a 13-item “No-Malignancy” checklist based on the selection strategy proposed by Suerink et al.(2019). We modified one item in the No-Malignancy checklist by replacing “Atypical CALMs,” with “One or more CALMs”, and adding the follow-up question: “were CALMs noted to be atypical?” as defined by irregular borders and/or different degrees of pigmentation. If the provider answered ‘No’ for “Atypical CALMs” the Integumentary/Skin section of the note was reviewed to ascertain the presence or absence of typical-appearing CALMs. Patients diagnosed with neoplasms including low-grade glioma, optic pathway glioma, plexiform neurofibroma, or cutaneous neurofibroma were evaluated using the No-Malignancy checklist. Patients were categorized as “Unknown” for meeting CMMRD evaluation criteria if either the presence of a diagnostic NF1 feature or the parent’s NF1 status was recorded as “Unknown”.

### Data collection

Data were extracted from the medical record into REDCap, a secure database (Harris 2019, Harris 2009). Each patient encounter generated a separate REDCap entry; thus, individuals seen in both Phase 1 and Phase 2 had distinct entries to maintain phase-specific data. Recorded data included demographics and results of genetic testing. A patient being evaluated in the NF Program for the first time was considered a “new” patient, compared to a “return” patient who had previously been evaluated by any provider in the NF Program. Sibling relationships were tracked by documenting the primary phone number listed in the chart. For Phase 1 encounters, the full consultation note was reviewed and the CMMRD checklist was completed retrospectively. In Phase 2, the provider-completed checklist responses were transcribed directly into REDCap without re-abstracting the clinical narrative.

### Data analysis

All statistical analyses were performed by a biostatistician (LW). Primary outcomes included the average number of “Unknown” responses per checklist, the proportion of patients fulfilling CMMRD evaluation criteria, and the rate of referral to genetics. Comparisons between Phase 1 and Phase 2 were conducted using a Fisher’s exact test for categorical variables due to the potentially small numbers of patients in any one crosstab cell. In addition to comparing demographic and visit-related variables within each phase and in the combined sample, we explored whether endorsement of each individual checklist item was associated with receiving a genetics referral. A t-test was employed to test whether age differed between Phase 1 and Phase 2 patients or between those receiving a genetics referral. To maintain independent samples, patients seen in both phases were analyzed only in the Phase 2 cohort. Six ethnicities were represented in the sample, however, only three ethnicities (Black, Hispanic, and White) had sufficient numbers to be compared between Phase 1 and 2. Means, standard errors (SE), and percentages are provided. SAS v 9.4 was utilized for all analyses.

## Results

### Patient demographics

**Fig. 1:**
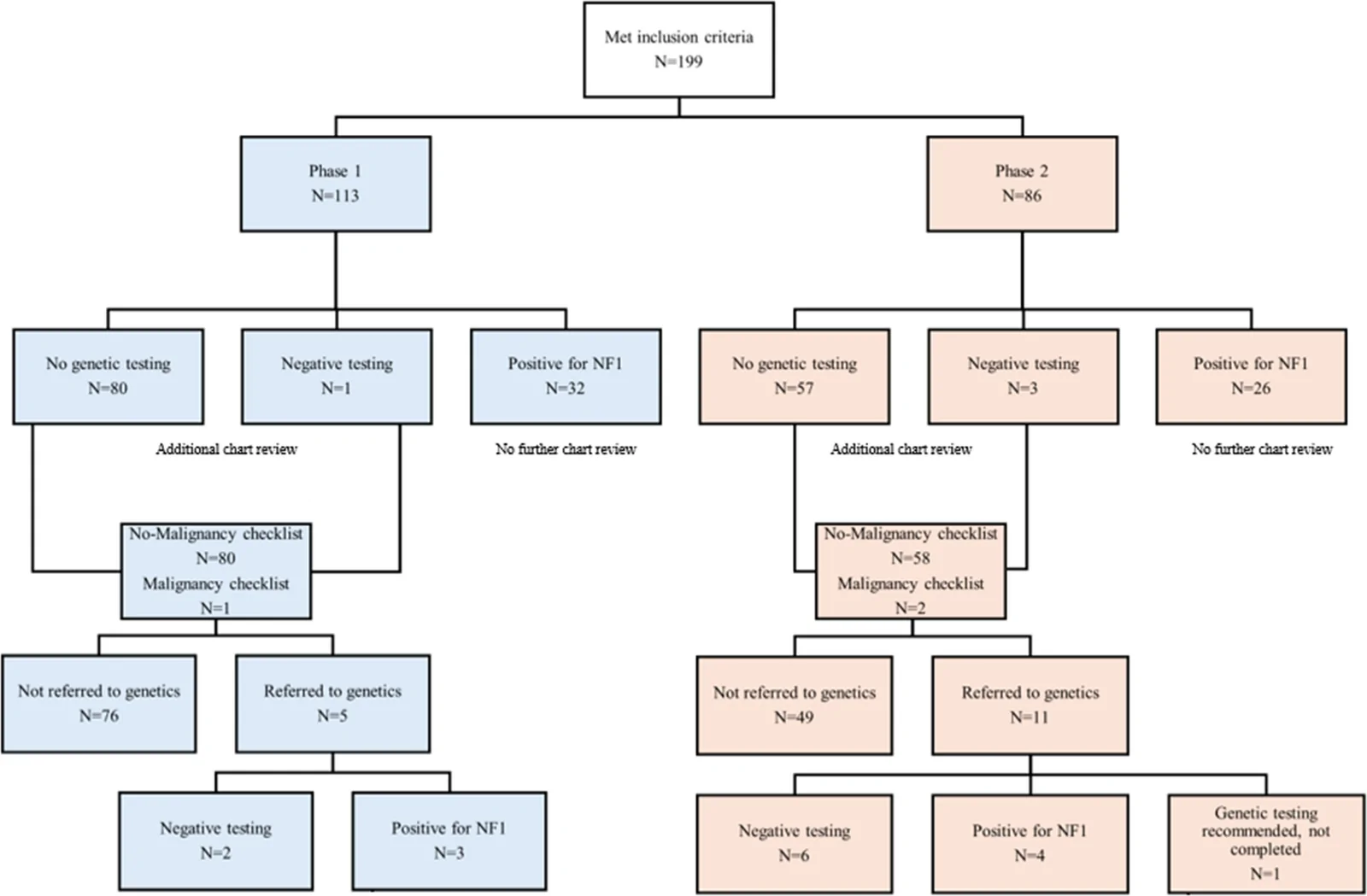
Flow chart of patients included in Phase 1 and Phase 2 and genetic testing outcomes. Footnote: Positive results indicate a pathogenic or likely pathogenic variant identified in the *NF1* gene. Negative results indicate a variant of uncertain significance identified in the *NF1* gene or negative testing. NF1 = neurofibromatosis type 1

Phase 1 included 113 patients (Fig. 1). Of these, 33 (29%) patients had undergone *NF1* gene testing prior to the NF clinic evaluation; 32 were found to carry an *NF1* PV, and one patient had negative germline testing. No patients with *SPRED1* PVs were identified. Phase 2 included 86 patients (Fig. 1), with 29 (34%) receiving *NF1* gene testing prior to their appointment; 26 patients tested positive and the remaining 3 tested negative.

The percentage of new patients was higher in Phase 2 (27%, *n* = 16) than in Phase 1 (12%, *n* = 10). Among all patients evaluated with either checklist, 80% (*n* = 113/141) had at least one neoplasm (plexiform neurofibroma = 65, cutaneous neurofibroma = 45, optic glioma = 31, low-grade glioma = 13, other = 14). Three patients were evaluated using the Malignancy checklist for diagnoses including rhabdomyosarcoma, MPNST, and low-grade sarcoma.

Both Phase 1 and 2 consisted of 55% female patients and 45% male patients (Table 1). The distribution of patients of Black, Hispanic, or White ethnicity did not differ significantly between phases (Fisher’s exact *p* = 0.97). Ages ranged from 11 months to 23 years, with similar mean ages in Phase 1 (10.80 years, SE = 0.50) and Phase 2 (10.66 years, SE = 0.56). The majority (93%, *n* = 171) of patients did not have a family member included in the study; however, eight families of two individuals (4%) and four families of three individuals (2%) were identified.

**Table 1:** Patient demographics within Phase 1 and Phase 2

		**Phase 1 (N = 113)**	**Phase 2 (N = 86)**	
		**N (%)**	**N (%)**	**P-value**
**Sex**	Females	62 (55%)	47 (55%)	1.0
	Males	51 (45%)	39 (45%)	
**Ethnicity**	White/Caucasian	80 (70.8%)	67 (78%)	0.97
	Hispanic	12 (10.6%)	10 (12%)	
	African American/Black	11 (9.7%)	8 (9%)	
	Two or more	8 (7.1%)	0	
	Asian	2 (1.8%)	0	
	Unknown	0	1 (1%)	
**Visit type**	New patients	10 (12%)	16 (27%)	0.047
	Return patients	71 (88%)	44 (73%)	
**Pre-visit NF1 testing**	No	3 (3%)	57 (66%)	
	Yes	33 (29%)	29 (34%)	
	Unknown	77 (68%)	0	
		**Phase 1 (N = 81)**	**Phase 2 (N = 60)**	
**Checklist used**	Malignancy checklist	1 (1%)	2 (3%)	
	No-Malignancy checklist	80 (99%)	58 (97%)	
**Diagnosed with a neoplasm**	No	22 (27.16%)	6 (10%)	
	Yes	59 (72.84%)	54 (90%)	

NF1 = neurofibromatosis type 1

### Checklist responses

**Fig. 2:**
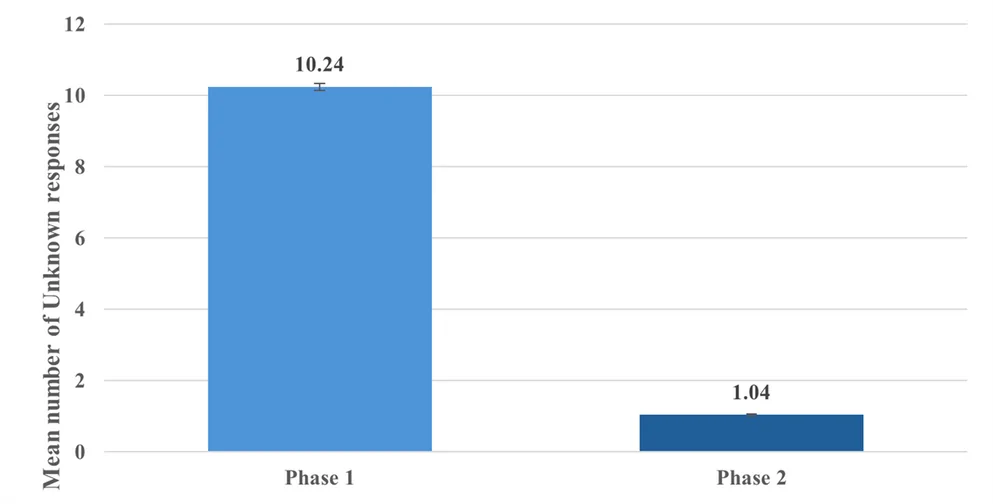
Mean number of Unknown responses per No-Malignancy checklist

The No-Malignancy checklist contained 13 items with Yes, No, or Unknown response options. In Phase 1, the average number of Unknown responses per patient was 10.24 (SE = 0.10). In contrast, the average number of Unknown responses in Phase 2 was substantially reduced to 1.04 (SE = 0.03; *p* < 0.0001) (Fig. 2).

### Genetic testing and genetic counseling

**Fig. 3:**
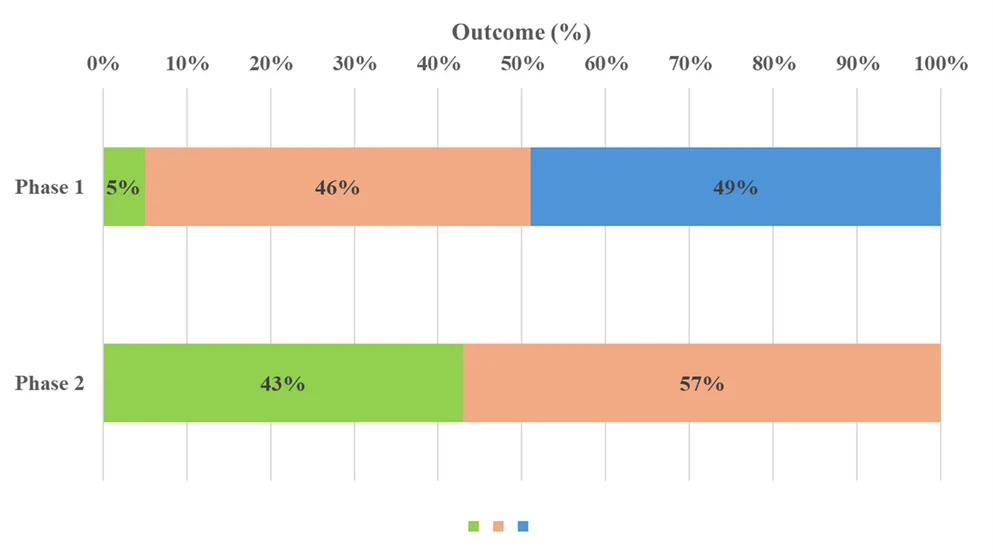
Distribution of patients who met criteria for CMMRD evaluation using published criteria. CMMRD = constitutional mismatch repair deficiency

**Fig. 4:**
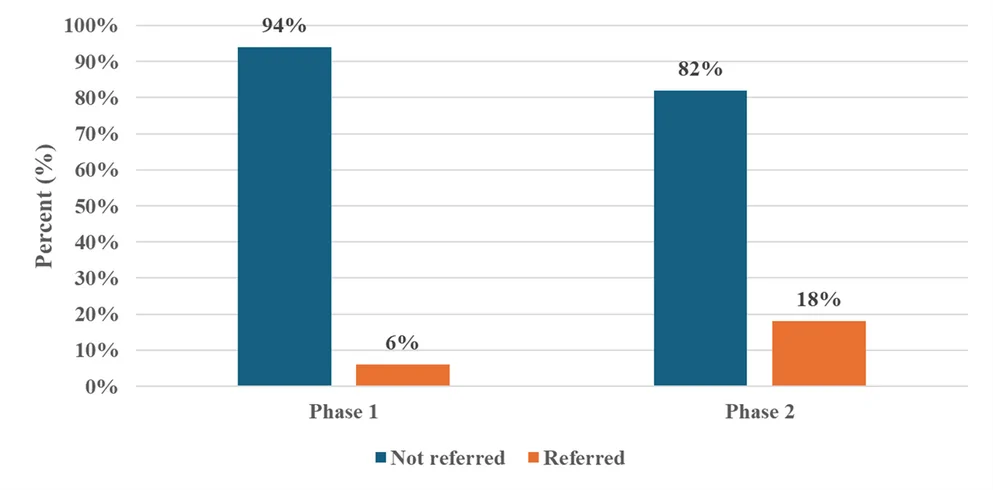
Distribution of all patients referred for genetic evaluation following their NF clinic visit. Includes patients who did and did not meet criteria for CMMRD evaluation. NF = neurofibromatosis; CMMRD = constitutional mismatch repair deficiency

A greater proportion of Phase 2 patients met criteria for CMMRD evaluation (43%; *n* = 26) compared to Phase 1 (5%; *n* = 4; Fisher’s exact *p* < 0.0001) (Fig. 3). Compared with Phase 1, patients in Phase 2 were significantly more likely to be referred to genetics (*p* = 0.032) following their NF clinic evaluation. Overall, 6% (*n* = 5) of Phase 1 patients were referred to genetics compared to 18% (*n* = 11) of Phase 2 patients (Fig. 4).

Of the four patients in Phase 1 who met criteria, none were referred to genetics, whereas 5 of the 26 patients in Phase 2 (19%) who met criteria received genetics referrals. 46% (*n* = 37) of Phase 1 patients and 57% (*n* = 34) of Phase 2 patients did not meet criteria. Nearly half of Phase 1 patients (49%; *n* = 40) could not be classified because of insufficient information, while all Phase 2 patients had complete data to fully assess CMMRD evaluation criteria.

In total, most patients in our study (61%, *n* = 121/199) did not undergo genetic testing before or after their NF clinic visit. Nearly all patients referred to genetics for further evaluation following their NF clinic visit subsequently received genetic testing (94%, *n* = 15/16), with DNA analysis only. The most frequently ordered test was single-gene *NF1* analysis (69%, *n* = 11), which identified 6 patients with P/LP *NF1* variants. Four patients received an *NF1/SPRED1* gene panel, with one P/LP *NF1* variant; whole genome sequencing was recommended for one patient, and this testing has not yet been completed. None of the patients with negative *NF1* gene testing had siblings in the study or siblings with NF1 features. None of the genetic testing completed included follow-up analysis of the DNA MMR genes.

### Factors impacting referral to genetics

In the combined Phase 1 + 2 sample, new patients were more likely to be referred to genetics than return patients (Fisher’s *p* < 0.001), which was primarily driven by the association in Phase 2 (Fisher’s *p* = 0.0005; Phase 1 *p* = 0.11). Several criteria within the No-Malignancy checklist were associated with a lower likelihood of genetics referral when marked as Unknown. Specifically, patients with Unknown responses for parent with NF1 features (*p* = 0.015), consanguinity (*p* = 0.027), or sibling with NF1 features (*p* = 0.048) were less likely to be referred to genetics than those with a No response to these items. Unknown responses to having a parent with Lynch syndrome, relative with cancer, CALMs, hypopigmented patch, pilomatricoma, agenesis of corpus callosum (ACC), or vascular malformation were associated with a tendency toward lower referral rates as compared to those with a No response, although these trends were not statistically significant (0.05 < *p* < 0.08). Within individual phases, none of these criteria were associated with referral patterns (all *p* > 0.09).

There was no correlation between meeting criteria for CMMRD evaluation and being referred to genetics in Phase 1 (*p* = 0.37) or Phase 2 (*p* = 1.00). Similarly, there was no association of genetics referral with patient age or negative *NF1/SPRED1* testing (both *p* > 0.5).

## Discussion

Phenotypic overlap between NF1 and CMMRD makes establishing an accurate diagnosis challenging. Previous work by Wimmer et al. (2014) and Suerink et al. (2019) introduced structured checklists to guide clinicians in identifying patients who may warrant evaluation for CMMRD for those with and without cancer, respectively.

In our study, implementing these published checklists directly into NF clinic notes substantially reduced ambiguity in documenting patients’ clinical features and determining appropriateness of CMMRD evaluation. Although some “Unknown” checklist responses persisted in Phase 2, they were markedly less common than in Phase 1. The resulting increase in unambiguous responses was essential for assessing whether patients met established criteria for CMMRD evaluation. All Phase 2 clinical notes included sufficient detail to determine if CMMRD evaluation was indicated, reflecting the improved clarification achieved through checklist implementation.

The CMMRD evaluation criteria (Suerink 2019) includes several family history data points. Documentation of parental NF1 features, a criterion required in the No-Malignancy checklist adapted from Suerink et al. (2019), was frequently missing in Phase 1 clinical notes. Consanguinity was commonly recorded as unknown in both phases, which contributed to the average of approximately one Unknown response per checklist in Phase 2. These findings underscore the essential role of obtaining and documenting a complete family history to help distinguish between NF1 and CMMRD.

Patients who were new to the NF clinic were more likely to be referred to genetics for further evaluation than returning patients. We speculate new patients may undergo a more comprehensive initial assessment, including a detailed review of their medical and family histories and evaluations by multiple specialists, including genetic counselors.

The study did not identify a relationship between meeting criteria for CMMRD evaluation and genetics referral. Because checklist scoring was completed retroactively, NF providers were unaware during the clinical encounter whether their patient met criteria. The scoring algorithms for the checklists were not included in the EMR dot phrase itself, representing a barrier to a clinician easily interpreting whether a positive screen had been triggered. These factors likely contributed to the lack of genetics referrals for those who met CMMRD criteria.

Over half of the patients in our study did not undergo genetic testing either before or after their NF clinic visit. Several patients received nondiagnostic *NF1* single-gene testing that did not include *SPRED1*, long-read sequencing, or RNA-based testing. It is possible patients in those populations could harbor a pathogenic *SPRED1* variant or a deep intronic *NF1* variant that would not have been detected with commercial DNA-only testing (Evans 2016). Among those who received testing, 15% (*n* = 12/78) had negative *NF1* results. These findings are comparable to another study that found 133 out of 240 (55%) individuals with features of NF1 did not receive genetic testing, and 25 out of 107 (23%) who did undergo *NF1* gene testing had nondiagnostic results (Hicks 2021). It is possible patients in our study who did not receive genetic testing or received nondiagnostic genetic testing may have a deep intronic *NF1* variant that was not detected by DNA-only testing or may harbor a *SPRED1* variant. Alternatively, it is possible some patients could have a different diagnosis, such as CMMRD.

Given the results of our study and the variety of potential diagnoses, current testing strategies should be re-evaluated, including increased testing for *NF1/SPRED1* with reflex to CMMRD testing for those with nondiagnostic results, as suggested by Colas et al.(2024). Historically, an NF1 diagnosis has relied on clinical criteria. Embedding genetic counselors within our multidisciplinary clinic has increased the number of genetic tests ordered, although most patients have not completed genetic testing. Ongoing investigations are aimed at identifying barriers to genetic counseling and genetic testing uptake.

A previous study estimated that approximately 0.4% of children suspected of having NF1, but who test negative for pathogenic *NF1* variants, have CMMRD (Suerink 2019). Although no patients in our study were found to have CMMRD, this likely reflects a limited sample size and the current genetic testing approach, which does not typically include follow-up analysis of the DNA MMR genes. This study highlights the need to pursue expanded genetic testing in patients who meet CMMRD evaluation criteria and/or patients with nondiagnostic *NF1* results to ensure accurate diagnosis and appropriate medical management.

### Limitations

A retrospective chart review introduces the possibility of human error and variability during data collection. We implemented measures including concordance review and a rigorous standard for data extraction to minimize these risks. The generalizability of this study may be limited, as data were collected from a clinic within one institution. This study is not generalizable to patients with a history of malignancy, as insufficient data were available on this population to run isolated statistical analyses.

## Conclusion

This study found implementing a clinical checklist for CMMRD evaluation reduced ambiguity in documenting both patient phenotype and appropriateness for further assessment. This provides a process to assess which patients need further evaluation, with the goal of identifying individuals with a diagnosis of CMMRD. However, although a greater proportion of patients met criteria for CMMRD evaluation after checklist implementation (Phase 2), in our study, this did not translate into increased genetics referrals and no patient in this study received genetic testing for CMMRD. This suggests a potential gap in provider awareness or follow-through of patients who met evaluation criteria or patients who had nondiagnostic *NF1* gene testing. Our study supports the importance of establishing clear workflows after nondiagnostic *NF1/SPRED1* genetic testing which may include reflex analysis of the DNA MMR genes, and highlights the opportunity to increase provider awareness of CMMRD through a streamlined approach to assess these patients.

## Supplementary Information

Supplementary Material 1.

## Data Availability

Data reported in this paper is available from the corresponding author upon reasonable request in compliance with IRB regulations.
