# Supplementary material for: Effect of implementation of a clinical checklist on the evaluation of constitutional mismatch repair deficiency in a neurofibromatosis clinic
Source: J Community Genet. 2026 Oct 30;17(6):134. doi: 10.1007/s12687-026-00967-8 (PMC13719907; doi:10.1007/s12687-026-00967-8)
Supplement: Supplementary file 1 [file 12687_2026_967_MOESM1_ESM.docx]

SUPPLEMENTAL LIST 1 Malignancy checklist and scoring system

- Questions 1-5: at least one answer must be 'Yes'
- Questions 6-16: optional
- For 'Yes' responses, all points were added
- The patient met criteria if they had 3 or more points

1. Any of the following cancers < 25yo: colorectal, endometrial, small bowel, ureter, renal pelvis, biliary tract, stomach, bladder (3 points)
2. Multiple bowel adenomas or a single high-grade dysplasia adenoma < 25yo (3 points)
3. WHO grade III or IV glioma < 25yo (2 points)
4. NHL of T-cell lineage or sPNET < 18yo (2 points)
5. Any malignancy < 18yo (1 point)
6. Clinical sign of NF1 and/or at least 2 hyperpigmented and/or hypopigmented skin alterations >1cm (2 points)
7. Any atypical CALMs (no points attached)
8. Lynch syndrome in a 1st or 2nd degree relative (2 points)
9. Any of the following cancers < 60yo in a 1st, 2nd, or 3rd degree relative: colorectal, endometrial, small bowel, ureter, renal pelvis, biliary tract, stomach, bladder (1 point)
10. Sibling with a high-grade glioma, sPNET, NHL, colorectal, endometrial, small bowel, ureter, renal pelvis, biliary tract, stomach, or bladder cancer (2 points)
11. Sibling with any type of childhood malignancy (1 point)
12. More than one pilomatricoma in the patient (2 points)
13. One pilomatricoma in the patient (1 point)
14. Agenesis of the corpus callosum or non-therapy-induced cavernoma in the patient (1 point)
15. Consanguineous parents (1 point)
16. Deficiency/reduced levels of IgG2/4 and/or IgA (1 point)

SUPPLEMENTAL LIST 2 No-Malignancy checklist and scoring system

- Question 1: answer must be 'Yes'
- Question 2: answer must be 'No'
- Questions 3-13: at least one answer must be 'Yes'
- Patient met criteria if all the above requirements were met

1. The presence of at least one diagnostic NF1 feature
2. Does either parent have diagnostic NF1 features?
3. Consanguineous parents
4. Genetic diagnosis of Lynch syndrome in one, or both, of the parental families
5. Sibling with one or more diagnostic NF1 signs
6. Sibling with any type of childhood malignancy
7. 1st or 2nd degree relative with any of the following cancers < 60yo: colorectal, endometrial, ovarian, gastric, small bowel, bile duct or gall bladder, pancreatic, or urothelial
8. Presence of one or more CALMs
9. Hypopigmented skin patches
10. One or more pilomatricomas
11. Agenesis of the corpus callosum
12. Non-therapy-induced cavernoma
13. Multiple developmental vascular abnormalities (cerebral venous angiomas) in separate regions of the brain (excluding moyamoya disease)

**Supplemental Table 1** Patient demographics within Phase 1 and Phase 2

|  |  | **Phase 1 (N = 113)** | **Phase 2 (N = 86)** |  |
| --- | --- | --- | --- | --- |
|  |  | **N (%)** | **N (%)** | **P-value** |
| **Sex** | Females | 62 (55%) | 47 (55%) | 1.0 |
|  | Males | 51 (45%) | 39 (45%) |  |
| **Ethnicity** | White/Caucasian | 80 (70.8%) | 67 (78%) | 0.97 |
|  | Hispanic | 12 (10.6%) | 10 (12%) |  |
|  | African American/Black | 11 (9.7%) | 8 (9%) |  |
|  | Two or more | 8 (7.1%) | 0 |  |
|  | Asian | 2 (1.8%) | 0 |  |
|  | Unknown | 0 | 1 (1%) |  |
| **Visit type** | New patients | 10 (12%) | 16 (27%) | 0.047 |
|  | Return patients | 71 (88%) | 44 (73%) |  |
| **Pre-visit NF1 testing** | No | 3 (3%) | 57 (66%) |  |
|  | Yes | 33 (29%) | 29 (34%) |  |
|  | Unknown | 77 (68%) | 0 |  |
|  |  | **Phase 1 (N = 81)** | **Phase 2 (N = 60)** |  |
| **Checklist used** | Malignancy checklist | 1 (1%) | 2 (3%) |  |
|  | No-Malignancy checklist | 80 (99%) | 58 (97%) |  |
| **Diagnosed with a neoplasm** | No | 22 (27.16%) | 6 (10%) |  |
|  | Yes | 59 (72.84%) | 54 (90%) |  |

NF1 = neurofibromatosis type 1
